# *N*-(4-Methyl­phen­yl)succinamic acid

**DOI:** 10.1107/S1600536810053298

**Published:** 2010-12-24

**Authors:** B. S. Saraswathi, Sabine Foro, B. Thimme Gowda, Hartmut Fuess

**Affiliations:** a Department of Chemistry, Mangalore University, Mangalagangotri 574 199, Mangalore, India; b Institute of Materials Science, Darmstadt University of Technology, Petersenstrasse 23, D-64287 Darmstadt, Germany

## Abstract

In the title compound, C_11_H_13_NO_3_, the conformations of the N—H and C=O bonds in the amide segment are *anti* to each other. Further, the conformations of the amide and carbonyl O atoms of the acid segment are also *anti* to the adjacent –CH_2_ groups. The C=O and O—H bonds of the acid group are *syn* to each other. In the crystal, mol­ecules are packed into infinite chains along the *b* axis through inter­molecular N—H⋯O and O—H⋯O hydrogen bonds.

## Related literature

For background to our study of the effect of ring and side-chain substitution on the solid state geometry of anilides, see: Gowda *et al.* (2009 ▶, 2010*a* ▶,*b* ▶). For modes of inter­linking carb­oxy­lic acids by hydrogen bonds, see: Leiserowitz (1976 ▶). The packing of mol­ecules involving dimeric hydrogen-bonded association of each carboxyl group with a centrosymmetrically related neighbor has also been observed, see: Jagannathan *et al.* (1994 ▶).
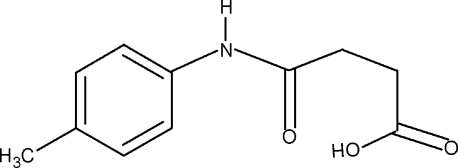


## Experimental

### 

#### Crystal data

C_11_H_13_NO_3_
*M* *_r_* = 207.22
Triclinic, 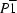

*a* = 4.960 (1) Å
*b* = 8.090 (2) Å
*c* = 13.893 (2) Å
α = 83.52 (2)°
β = 80.08 (2)°
γ = 78.15 (1)°
*V* = 535.70 (19) Å^3^
*Z* = 2
Cu *K*α radiation
μ = 0.78 mm^−1^
*T* = 299 K
0.55 × 0.25 × 0.08 mm

#### Data collection

Enraf–Nonius CAD-4 diffractometer
Absorption correction: ψ scan (North *et al.*, 1968 ▶) *T*_min_ = 0.674, *T*_max_ = 0.940
2515 measured reflections
1888 independent reflections
1533 reflections with *I* > 2σ(*I*)
*R*_int_ = 0.024
3 standard reflections every 120 min  intensity decay: 1.0%

#### Refinement

*R*[*F* ^2^ > 2σ(*F* ^2^)] = 0.064
*wR*(*F* ^2^) = 0.194
*S* = 1.06
1888 reflections
143 parameters
2 restraints
H atoms treated by a mixture of independent and constrained refinement
Δρ_max_ = 0.24 e Å^−3^
Δρ_min_ = −0.33 e Å^−3^

### 

Data collection: *CAD-4-PC* (Enraf–Nonius, 1996 ▶); cell refinement: *CAD-4-PC*; data reduction: *REDU4* (Stoe & Cie, 1987 ▶); program(s) used to solve structure: *SHELXS97* (Sheldrick, 2008 ▶); program(s) used to refine structure: *SHELXL97* (Sheldrick, 2008 ▶); molecular graphics: *PLATON* (Spek, 2009 ▶); software used to prepare material for publication: *SHELXL97*.

## Supplementary Material

Crystal structure: contains datablocks I, global. DOI: 10.1107/S1600536810053298/ds2081sup1.cif

Structure factors: contains datablocks I. DOI: 10.1107/S1600536810053298/ds2081Isup2.hkl

Additional supplementary materials:  crystallographic information; 3D view; checkCIF report

## Figures and Tables

**Table 1:** Hydrogen-bond geometry (Å, °)

*D*—H⋯*A*	*D*—H	H⋯*A*	*D*⋯*A*	*D*—H⋯*A*
N1—H1*N*⋯O1^i^	0.84 (2)	2.15 (2)	2.979 (2)	175 (3)
O3—H3*O*⋯O2^ii^	0.84 (2)	1.84 (2)	2.681 (3)	171 (4)

Symmetry codes: (i) 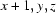; (ii) 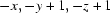.

**Fig. 1.**
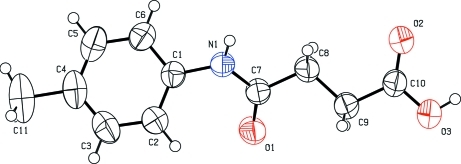
Molecular structure of the title compound, showing the atom labelling scheme. The displacement ellipsoids are drawn at the 50% probability level. The H atoms are represented as small spheres of arbitrary radii.

**Fig. 2.**
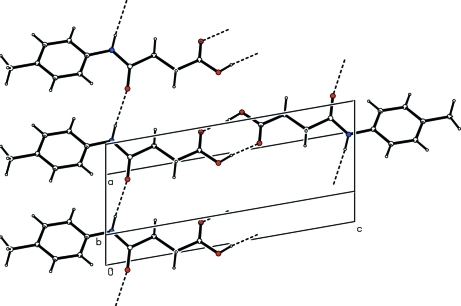
Molecular packing of the title compound with hydrogen bonding shown as dashed lines.

## supplementary crystallographic information

### Comment

In the present work, as a part of studying the effect of ring and side chain substitutions on the solid state geometry of anilides (Gowda *et al.*, 2009, 2010**a*, b*), the crystal structure of *N*-(4-methylphenyl)- succinamic acid (I) has been determined. The conformations of N—H and C=O bonds in the amide segment are *anti* to each other. The conformation of the amide oxygen and the carbonyl oxygen of the acid segment are also *anti* to each other, similar to that observed in *N*-(4-Chlorophenyl)succinamic acid (II) (Gowda *et al.*, 2009) and *N*-(2-methylphenyl)-succinamic acid (III)(Gowda *et al.*, 2010*b*). but contrary to the *syn* conformation observed in *N*-(3-methylphenyl)-succinamic acid (IV) (Gowda *et al.*, 2010*a*).

Further, the conformation of both the C=O bonds are *anti* to the H atoms of their adjacent –CH_2_ groups (Fig. 1) and the C=O and O—H bonds of the acid group are in *syn* position to each other, similar to that observed in (II), (III) and (IV).

The N—H···O and O—H···O intermolecular hydrogen bonds pack the molecules into infinite chains in the structure (Table 1, Fig.2).

The modes of interlinking carboxylic acids by hydrogen bonds is described elsewhere (Leiserowitz, 1976). The packing of molecules involving dimeric hydrogen bonded association of each carboxyl group with a centrosymmetrically related neighbor has also been observed (Jagannathan *et al.*, 1994).

### Experimental

The solution of succinic anhydride (0.01 mole) in toluene (25 ml) was treated dropwise with the solution of *p*-toluidine (0.01 mole) also in toluene (20 ml) with constant stirring. The resulting mixture was stirred for about one h and set aside for an additional hour at room temperature for completion of the reaction. The mixture was then treated with dilute hydrochloric acid to remove the unreacted *p*-toluidine. The resultant solid *N*-(4-methylphenyl)- succinamic acid was filtered under suction and washed thoroughly with water to remove the unreacted succinic anhydride and succinic acid. It was recrystallized to constant melting point from ethanol. The purity of the compound was checked by elemental analysis and characterized by its infrared and NMR spectra.

The plate like colorless single crystals used in X-ray diffraction studies were grown in ethanolic solution by slow evaporation at room temperature.

### Refinement

The H atoms of the NH group and OH group were located in a difference map and later restrained to the distance N—H = 0.86 (2) Å and O—H = 0.82 (2) Å. The other H atoms were positioned with idealized geometry using a riding model [C—H = 0.93–0.97 Å]. All H atoms were refined with isotropic displacement parameters (set to 1.2 times of the *U*_eq_ of the parent atom).

### Crystal data

**Table:**

C_11_H_13_NO_3_	*Z* = 2
*M**_r_* = 207.22	*F*(000) = 220
Triclinic, *P*1	*D*_x_ = 1.285 Mg m^−^^3^
Hall symbol: -P 1	Cu *K*α radiation, λ = 1.54180 Å
*a* = 4.960 (1) Å	Cell parameters from 25 reflections
*b* = 8.090 (2) Å	θ = 6.3–21.3°
*c* = 13.893 (2) Å	µ = 0.78 mm^−^^1^
α = 83.52 (2)°	*T* = 299 K
β = 80.08 (2)°	Plate, colourless
γ = 78.15 (1)°	0.55 × 0.25 × 0.08 mm
*V* = 535.70 (19) Å^3^	

### Data collection

**Table:**

Enraf–Nonius CAD-4 diffractometer	1533 reflections with *I* > 2σ(*I*)
Radiation source: fine-focus sealed tube	*R*_int_ = 0.024
graphite	θ_max_ = 66.8°, θ_min_ = 3.2°
ω scans	*h* = −1→5
Absorption correction: ψ scan (North *et al.*, 1968)	*k* = −9→9
*T*_min_ = 0.674, *T*_max_ = 0.940	*l* = −16→16
2515 measured reflections	3 standard reflections every 120 min
1888 independent reflections	intensity decay: 1.0%

### Refinement

**Table:**

Refinement on *F*^2^	Primary atom site location: structure-invariant direct methods
Least-squares matrix: full	Secondary atom site location: difference Fourier map
*R*[*F*^2^ > 2σ(*F*^2^)] = 0.064	Hydrogen site location: inferred from neighbouring sites
*wR*(*F*^2^) = 0.194	H atoms treated by a mixture of independent and constrained refinement
*S* = 1.06	*w* = 1/[σ^2^(*F*_o_^2^) + (0.1156*P*)^2^ + 0.1448*P*] where *P* = (*F*_o_^2^ + 2*F*_c_^2^)/3
1888 reflections	(Δ/σ)_max_ = 0.017
143 parameters	Δρ_max_ = 0.24 e Å^−^^3^
2 restraints	Δρ_min_ = −0.33 e Å^−^^3^

### Special details

**Table:**

Geometry. All e.s.d.'s (except the e.s.d. in the dihedral angle between two l.s. planes) are estimated using the full covariance matrix. The cell e.s.d.'s are taken into account individually in the estimation of e.s.d.'s in distances, angles and torsion angles; correlations between e.s.d.'s in cell parameters are only used when they are defined by crystal symmetry. An approximate (isotropic) treatment of cell e.s.d.'s is used for estimating e.s.d.'s involving l.s. planes.
Refinement. Refinement of *F*^2^ against ALL reflections. The weighted *R*-factor *wR* and goodness of fit *S* are based on *F*^2^, conventional *R*-factors *R* are based on *F*, with *F* set to zero for negative *F*^2^. The threshold expression of *F*^2^ > σ(*F*^2^) is used only for calculating *R*-factors(gt) *etc*. and is not relevant to the choice of reflections for refinement. *R*-factors based on *F*^2^ are statistically about twice as large as those based on *F*, and *R*- factors based on ALL data will be even larger.

### Fractional atomic coordinates and isotropic or equivalent isotropic displacement parameters (Å^2^)

**Table:**

	*x*	*y*	*z*	*U*_iso_*/*U*_eq_	
C1	0.0934 (5)	0.7741 (3)	−0.07532 (16)	0.0507 (5)	
C2	−0.1340 (6)	0.8882 (3)	−0.10364 (19)	0.0650 (6)	
H2	−0.2788	0.9345	−0.0567	0.078*	
C3	−0.1439 (7)	0.9328 (4)	−0.2025 (2)	0.0786 (8)	
H3	−0.2988	1.0075	−0.2211	0.094*	
C4	0.0703 (7)	0.8692 (4)	−0.27428 (19)	0.0761 (8)	
C5	0.2991 (7)	0.7575 (4)	−0.2444 (2)	0.0802 (8)	
H5	0.4466	0.7135	−0.2912	0.096*	
C6	0.3100 (6)	0.7110 (3)	−0.14645 (19)	0.0677 (7)	
H6	0.4650	0.6363	−0.1279	0.081*	
C7	−0.0863 (4)	0.6981 (3)	0.09714 (16)	0.0538 (6)	
C8	0.0133 (5)	0.6313 (3)	0.19407 (17)	0.0603 (6)	
H8A	0.0707	0.5091	0.1952	0.072*	
H8B	0.1748	0.6782	0.1992	0.072*	
C9	−0.2087 (5)	0.6752 (3)	0.28162 (17)	0.0603 (6)	
H9A	−0.2574	0.7974	0.2830	0.072*	
H9B	−0.3747	0.6347	0.2745	0.072*	
C10	−0.1152 (5)	0.5994 (3)	0.37648 (17)	0.0588 (6)	
C11	0.0523 (11)	0.9182 (6)	−0.3816 (2)	0.1183 (15)	
H11A	0.0186	1.0394	−0.3932	0.142*	
H11B	−0.0976	0.8753	−0.3993	0.142*	
H11C	0.2245	0.8709	−0.4206	0.142*	
N1	0.1202 (4)	0.7230 (2)	0.02421 (14)	0.0533 (5)	
H1N	0.278 (4)	0.719 (3)	0.0386 (19)	0.064*	
O1	−0.3349 (3)	0.7206 (3)	0.08761 (13)	0.0727 (6)	
O2	0.1144 (4)	0.5108 (3)	0.38248 (14)	0.0853 (7)	
O3	−0.3025 (4)	0.6364 (3)	0.45282 (14)	0.0935 (8)	
H3O	−0.231 (8)	0.598 (5)	0.504 (2)	0.112*	

### Atomic displacement parameters (Å^2^)

**Table:**

	*U*^11^	*U*^22^	*U*^33^	*U*^12^	*U*^13^	*U*^23^
C1	0.0523 (12)	0.0537 (11)	0.0487 (11)	−0.0180 (9)	−0.0068 (9)	−0.0023 (9)
C2	0.0656 (15)	0.0657 (14)	0.0593 (14)	−0.0061 (11)	−0.0098 (11)	0.0021 (11)
C3	0.088 (2)	0.0785 (17)	0.0699 (18)	−0.0168 (14)	−0.0264 (15)	0.0148 (13)
C4	0.111 (2)	0.0768 (17)	0.0508 (14)	−0.0433 (16)	−0.0182 (14)	0.0068 (12)
C5	0.103 (2)	0.0806 (17)	0.0537 (15)	−0.0238 (16)	0.0073 (14)	−0.0088 (13)
C6	0.0662 (15)	0.0722 (15)	0.0590 (14)	−0.0096 (12)	0.0006 (11)	−0.0032 (11)
C7	0.0464 (12)	0.0669 (13)	0.0505 (12)	−0.0183 (10)	−0.0089 (9)	0.0014 (9)
C8	0.0447 (12)	0.0842 (16)	0.0522 (13)	−0.0163 (10)	−0.0102 (10)	0.0061 (11)
C9	0.0529 (13)	0.0755 (15)	0.0514 (13)	−0.0124 (11)	−0.0098 (10)	0.0031 (10)
C10	0.0568 (13)	0.0705 (14)	0.0477 (12)	−0.0142 (11)	−0.0052 (10)	0.0006 (10)
C11	0.188 (4)	0.126 (3)	0.0569 (19)	−0.068 (3)	−0.030 (2)	0.0139 (18)
N1	0.0414 (9)	0.0713 (12)	0.0485 (10)	−0.0151 (8)	−0.0080 (7)	−0.0007 (8)
O1	0.0438 (9)	0.1125 (15)	0.0629 (11)	−0.0234 (9)	−0.0138 (7)	0.0128 (9)
O2	0.0686 (12)	0.1219 (17)	0.0518 (10)	0.0074 (11)	−0.0100 (8)	0.0047 (10)
O3	0.0740 (13)	0.1372 (19)	0.0504 (11)	0.0077 (12)	−0.0024 (9)	0.0096 (11)

### Geometric parameters (Å, °)

**Table:**

C1—C6	1.383 (3)	C7—C8	1.518 (3)
C1—C2	1.386 (3)	C8—C9	1.514 (3)
C1—N1	1.419 (3)	C8—H8A	0.9700
C2—C3	1.387 (4)	C8—H8B	0.9700
C2—H2	0.9300	C9—C10	1.497 (3)
C3—C4	1.383 (5)	C9—H9A	0.9700
C3—H3	0.9300	C9—H9B	0.9700
C4—C5	1.390 (5)	C10—O2	1.225 (3)
C4—C11	1.513 (4)	C10—O3	1.302 (3)
C5—C6	1.378 (4)	C11—H11A	0.9600
C5—H5	0.9300	C11—H11B	0.9600
C6—H6	0.9300	C11—H11C	0.9600
C7—O1	1.237 (3)	N1—H1N	0.836 (17)
C7—N1	1.340 (3)	O3—H3O	0.844 (19)
			
C6—C1—C2	119.1 (2)	C7—C8—H8A	109.0
C6—C1—N1	117.9 (2)	C9—C8—H8B	109.0
C2—C1—N1	123.0 (2)	C7—C8—H8B	109.0
C1—C2—C3	119.5 (2)	H8A—C8—H8B	107.8
C1—C2—H2	120.2	C10—C9—C8	112.4 (2)
C3—C2—H2	120.2	C10—C9—H9A	109.1
C4—C3—C2	121.8 (3)	C8—C9—H9A	109.1
C4—C3—H3	119.1	C10—C9—H9B	109.1
C2—C3—H3	119.1	C8—C9—H9B	109.1
C3—C4—C5	117.8 (2)	H9A—C9—H9B	107.9
C3—C4—C11	120.7 (3)	O2—C10—O3	122.9 (2)
C5—C4—C11	121.4 (3)	O2—C10—C9	123.8 (2)
C6—C5—C4	120.8 (3)	O3—C10—C9	113.4 (2)
C6—C5—H5	119.6	C4—C11—H11A	109.5
C4—C5—H5	119.6	C4—C11—H11B	109.5
C5—C6—C1	120.8 (3)	H11A—C11—H11B	109.5
C5—C6—H6	119.6	C4—C11—H11C	109.5
C1—C6—H6	119.6	H11A—C11—H11C	109.5
O1—C7—N1	124.4 (2)	H11B—C11—H11C	109.5
O1—C7—C8	121.9 (2)	C7—N1—C1	126.48 (19)
N1—C7—C8	113.68 (19)	C7—N1—H1N	118.0 (19)
C9—C8—C7	112.7 (2)	C1—N1—H1N	115.1 (19)
C9—C8—H8A	109.0	C10—O3—H3O	108 (3)
			
C6—C1—C2—C3	1.9 (4)	O1—C7—C8—C9	−27.7 (3)
N1—C1—C2—C3	179.6 (2)	N1—C7—C8—C9	154.7 (2)
C1—C2—C3—C4	−1.4 (4)	C7—C8—C9—C10	176.27 (19)
C2—C3—C4—C5	0.1 (4)	C8—C9—C10—O2	−0.5 (4)
C2—C3—C4—C11	179.2 (3)	C8—C9—C10—O3	179.6 (2)
C3—C4—C5—C6	0.5 (4)	O1—C7—N1—C1	−1.7 (4)
C11—C4—C5—C6	−178.6 (3)	C8—C7—N1—C1	175.8 (2)
C4—C5—C6—C1	0.1 (4)	C6—C1—N1—C7	−146.1 (2)
C2—C1—C6—C5	−1.4 (4)	C2—C1—N1—C7	36.3 (3)
N1—C1—C6—C5	−179.1 (2)		

### Hydrogen-bond geometry (Å, °)

**Table:**

*D*—H···*A*	*D*—H	H···*A*	*D*···*A*	*D*—H···*A*
N1—H1N···O1^i^	0.84 (2)	2.15 (2)	2.979 (2)	175 (3)
O3—H3O···O2^ii^	0.84 (2)	1.84 (2)	2.681 (3)	171 (4)

Symmetry codes: (i) *x*+1, *y*, *z*; (ii) −*x*, −*y*+1, −*z*+1.
